# Agmatine Enhances Dorsal Raphe Serotonergic Neuronal Activity via Dual Regulation of 5-HT_1B_ and 5-HT_2A_ Receptors

**DOI:** 10.3390/ijms26073087

**Published:** 2025-03-27

**Authors:** Hande Özbaşak, Ruslan Paliokha, Roman Dekhtiarenko, Daniil Grinchii, Eliyahu Dremencov

**Affiliations:** Centre of Biosciences, Institute of Molecular Physiology and Genetics, Slovak Academy of Sciences, 840 05 Bratislava, Slovakia; hande.ozbasak@savba.sk (H.Ö.); ruslan.paliokha@savba.sk (R.P.); roman.dekhtiarenko@savba.sk (R.D.); daniil.grinchii@savba.sk (D.G.)

**Keywords:** neuronal nitric oxide (NO) synthase (nNOS), serotonin (5-HT) transporter (SERT), serotonin-1A/AB receptors (5-HT_1A/1B_), serotonin-2A/2B/2C receptors (5-HT_2A/2B/2C_), antidepressant drug response

## Abstract

Agmatine is a naturally occurring biogenic amine that acts primarily as an inhibitor of neuronal nitric oxide synthase (nNOS). Previous studies have shown that both acute and chronic agmatine administration induced anxiolytic and antidepressant-like effects in rodents. In the dorsal raphe nucleus (DRN), nitric oxide (NO) donors inhibit serotonergic (5-HT) neuronal activity, with the nNOS-expressing 5-HT neurons showing lower baseline firing rates than the non-nNOS expressing neurons. Our study aimed to test the hypothesis that the psychoactive effects of agmatine are mediated, at least in part, via a mechanism involving the stimulation of the DRN 5-HT neurons, as well as to assess the molecular pathway allowing agmatine to modulate the excitability of 5-HT neurons. Using extracellular in vivo electrophysiology, we demonstrated that both acute (1–3 mg/kg, i.v.) and chronic (40 mg/kg/day, i.p., 14 days) agmatine administration significantly increased the firing rate of DRN 5-HT neurons. Quantitative PCR (qPCR) analysis revealed that chronic agmatine treatment selectively upregulated the expression of serotonin-1B (5-HT_1B_) and serotonin-2A (5-HT_2A_) receptor mRNA in the DRN. Previous studies have shown that DRN 5-HT_2A_ receptor activation stimulates 5-HT neurons and produces antidepressant-like effects; our findings suggest that agmatine’s excitatory effect on DRN 5-HT neurons may be partially 5-HT_2A_ receptor-dependent. Given that modulation of the 5-HT neuronal firing activity is critical for the proper antidepressant efficacy, nNOS inhibitors can be potential antidepressants by their own and/or effective adjuncts to other antidepressant drugs.

## 1. Introduction

Agmatine, a naturally occurring biogenic amine, is synthesized through the amino acid L-arginine by the enzyme arginine decarboxylase. Since its discovery in mammalian brains in the 20th century, agmatine has garnered considerable scientific attention due to its diverse biological activities and therapeutic potential [1,2]. One of the primary biological functions of agmatine is the inhibition of nitric oxide (NO) synthase (NOS) [3,4], particularly, of the neuronal form (nNOS) of this enzyme [5]. Since NO is an important neuromodulator, and nNOS is its primary source within the central nervous system (CNS), this enzyme modulates multiple brain neurotransmitter systems [6]. Among the CNS neurotransmitter systems modulated by nNOS, there is a serotonergic (5-HT) system, playing a key role in the pathophysiology and treatment of depression and related stress-related anxiety and mood disorders [7].

It was reported that nNOS molecularly binds to and creates a heterodimer with the 5-HT transporter (SERT) [8]. The latter plays a key role in the regulation of the extracellular 5-HT concentrations in the dorsal raphe nucleus (DRN), a brain area containing the cell bodies of 5-HT-secreing neurons. Extracellular 5-HT negatively regulates the firing activity of 5-HT neurons via a mechanism primarily involving somatodendritic serotonin-1A (5-HT_1A_) autoreceptors. The 5-HT_1A_ autoreceptors-mediated inhibition of 5-HT neurons of the DRN is responsible, at least in part, for the delayed behavioral response and/or for the lack of adequate therapeutic response to antidepressant drugs, such as the selected serotonin (5-HT) reuptake inhibitors [7]. Consistently, molecules dissociating the nNOS–SERT dimerization were reported to have robust and rapid antidepressant-like effect [9]. This molecular interaction between nNOS and SERT represents a potential therapeutic target, as disruption of this complex has been shown to produce rapid antidepressant effects.

Agmatine, as a natural inhibitor of the NOS/nNOS, exhibits significant psychoactive effects through the modulation of 5-HT neurotransmission. Chronic agmatine treatment produces robust antidepressant-like behavioral effect in mice, accompanied by increased extracellular brain concentrations of 5-HT and glutamate along with elevated expression of neuroplasticity markers such as brain derived neurotrophic factor (BDNF) and synaptotagmin I [10]. Additional studies have confirmed both anxiolytic and antidepressant-like effects following chronic agmatine treatment [11,12]. Notably, even acute agmatine treatment demonstrates antidepressant-like effect and increases BDNF levels [13], with similar anxiolytic effects reported in mice [14]. The mechanism underlying these behavioral effects may be linked to agmatine’s influence on 5-HT neuronal activity, as previous studies have shown that DRN 5-HT neurons expressing nNOS exhibit lower spontaneous firing rates compared to non-NOS expressing neurons. This is further supported by the observation that the nitric oxide donor diethylamineNONOate directly inhibits 5-HT neuronal firing in vitro electrophysiology [15]. These findings suggest that agmatine’s psychoactive effects might be mediated through a disinhibition of serotonergic neurotransmission via nNOS inhibition.

Building on the previous findings suggesting NO’s role in serotonergic signaling, we aimed to investigate, using in vivo electrophysiology, both the acute and chronic effects of agmatine on 5-HT neuronal firing activity. We further investigated the potential molecular adaptations following chronic agmatine treatment by analyzing the expression of nNOS, serotonin transporter (SERT), and key 5-HT receptor subtypes (serotonin-1A/AB receptors (5-HT_1A/1B_) and serotonin-2A/2B/2C (5-HT_2A/2B/2C_)) in the rat dorsal raphe nucleus (DRN).

## 2. Results

### 2.1. Effects Acute and Chronic Agmatine Treatment on the Firing Activity of 5-HT Neurons in the DRN

**Figure 1:**
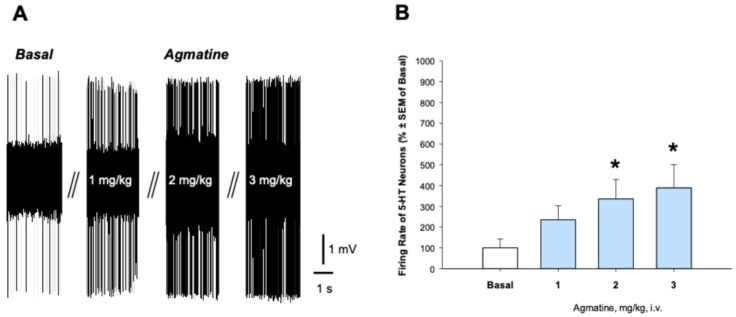
Effect of acute administration of agmatine on the firing activity of 5-HT neurons. (**A**) Representative recording from a selected DRN 5-HT neuron. (**B**) Summary effect from 6 neurons from 6 rats. * *p* < 0.05, Bonferroni post hoc test. Data are presented as mean ± SEM.

Figure 1 illustrates the effect of acute intravenous (i.v.) administration of agmatine, at the cumulative doses of 1–3 mg/kg, on the firing activity of 5-HT neurons of the DRN. Agmatine significantly and dose-dependently increased the firing rate of 5-HT neurons, comparing to their own basal firing activity (F_3,22_ = 4.45, analysis of variance for repeated measures: RM ANOVA). Bonferroni post hoc test confirmed a significant difference between the basal firing activity of 5-HT neurons and their firing activity after the administration of 2 and 3 mg/kg of agmatine (*p* < 0.05). We showed that the neuronal firing rate demonstrated a progressive increase correlated with increasing agmatine doses. The burst firing characteristics of 5-HT neurons, such as bursts’ frequence, percent of spikes occurring within the burst, and mean number of spikes in burst, were not altered after the acute agmatine administration.

### 2.2. Effects of Chronic Agmatine Treatment on the Firing Activity of 5-HT Neurons in the DRN

**Figure 2:**
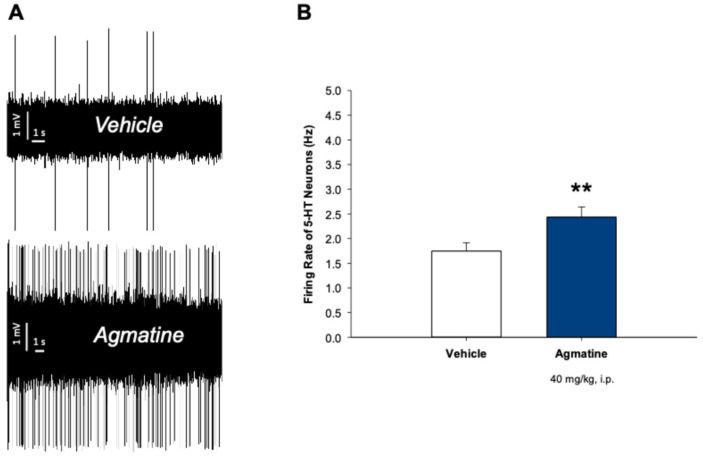
Effect of chronic treatment with agmatine on the firing activity of 5-HT neurons. (**A**) Representative recording from the selected DRN 5-HT neurons from the vehicle (up)- and agmatine (down)-treated rats. (**B**) Summary effect from 75 neurons from 6 vehicle- and 100 neurons from 6 agmatine-treated rats. ** *p* < 0.01, two-tailed Student’s *t*-test. Data are presented as mean ± SEM.

Figure 2 illustrates the effect of chronic intraperitoneal (i.p.) administration of agmatine on the firing activity of 5-HT neurons in the DRN. In this experiment, the rats received daily agmatine injections (40 mg/kg) for 14 consecutive days.

Chronic agmatine treatment led to the significant increase in the mean spontaneous firing rate of 5-HT neurons, compared to the vehicle-treated control animals (*p* < 0.01, two-tailed Student’s *t*-test). To further characterize the effects of chronic agmatine treatment, we examined the expression of nNOS, SERT, and serotonin receptor subtypes in the dorsal raphe nucleus (DRN) using a quantitative polymerase chain reaction (qPCR). The burst firing characteristics of 5-HT neurons, such as burst frequency and percent of spikes occurring within the burst, were not affected by the chronic agmatine treatment.

### 2.3. Effects of Chronic Agmatine Treatment on the nNOS and SERT mRNA Expression in the DRN

**Figure 3:**
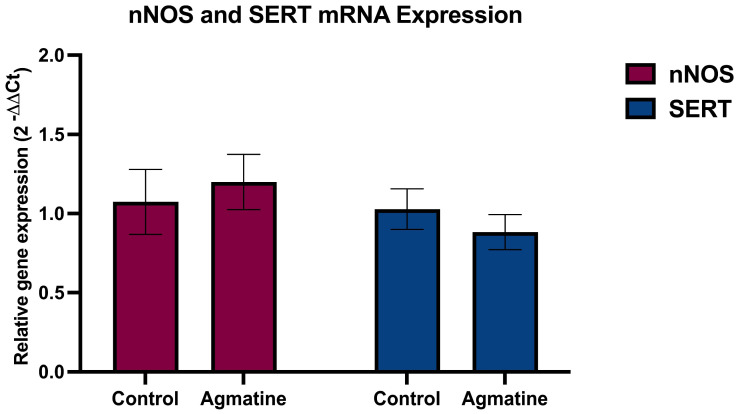
Relative mRNA expression of nNOS and SERT in the DRN of vehicle- and agmatine-treated rats. Data are presented as mean ± SEM. (*n* = 5 rats per group, two-tailed Student *t*-test; Mann–Whitney test, *p* > 0.05). For the raw fluorescence data, see Supplementary Materials.

Figure 3 presents the results of the RT-qPCR analysis examining the expression of mRNA encoding nNOS and SERT in the DRN. Contrary to the initial hypothesis, the chronic i.p. administration of agmatine (40 mg/kg/day for 14 days) did not induce statistically significant changes in the mRNA expression levels of either nNOS or SERT in the DRN (*p* > 0.05, two-tailed Student’s *t*-test).

### 2.4. Effects of Chronic Agmatine Treatment on the Expression of the DRN 5-HT Receptors Belonging to the 5-HT_1_ Subfamily

**Figure 4:**
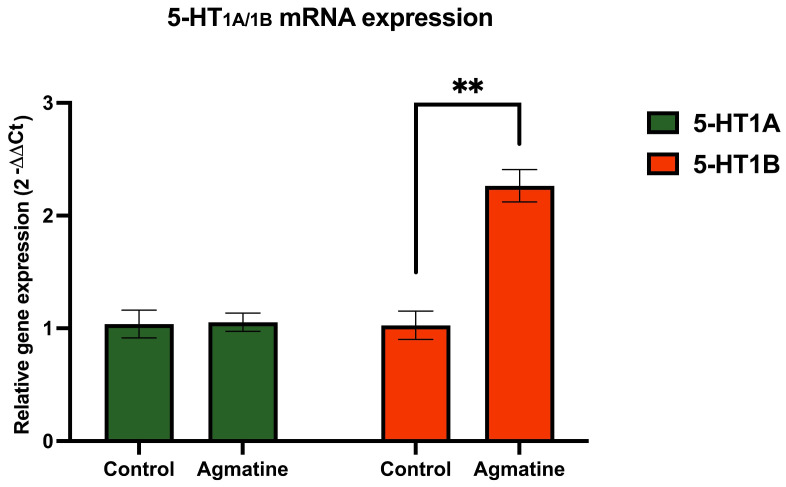
Relative mRNA expression of 5-HT_1A_ and 5-HT_1B_ receptors in the DRN of vehicle- and agmatine-treated rats. Significant upregulation of 5-HT_1B_ receptor mRNA expression was observed in agmatine-treated rats. Data are presented as mean ± SEM. (*n* = 5 rats per group, two-tailed Student *t*-test; Mann–Whitney test, ** *p* < 0.01). For the raw fluorescence data, see Supplementary Materials.

Figure 4 illustrates the effect of chronic agmatine treatment on the mRNA expression of 5-HT receptors belonging to the 5-HT_1_ subfamily in the DRN. The mRNA encoding the 5-HT_1A_ receptor in the agmatine-treated rats showed no statistical difference compared to the control group. In contrast, the chronic agmatine treatment led to a robust and statistically significant increase in the expression of the 5-HT_1B_ receptor encoding mRNA in the DRN (** *p* < 0.01, two-tailed Student’s *t*-test).

### 2.5. Effects of Chronic Agmatine Treatment on the Expression of the DRN 5-HT Receptors Belonging to the 5-HT_2_ Subfamily

**Figure 5:**
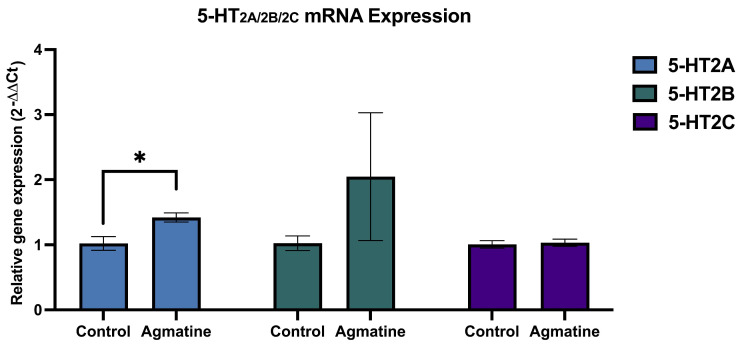
Relative mRNA expression of 5-HT_2A_, 5-HT_2B_, and 5-HT_2C_ in the DRN. Significant upregulation of 5-HT_2A_ receptor mRNA expression was observed in agmatine-treated rats. Data are presented as mean ± SEM. (*n* = 5 rats per group, two-tailed Student’s *t*-test; Mann–Whitney test, * *p* < 0.05). For the raw fluorescence data, see Supplementary Materials.

Figure 5 illustrates the effect of chronic agmatine treatment on the mRNA expression of serotonin-2A (5-HT_2A_), serotonin-2B (5-HT_2B_), and serotonin-2C (5-HT_2C_) receptors in the DRN.

Chronic agmatine led to the statistically significant increase in the expression of mRNA encoding 5-HT_2A_ receptor in the DRN, compared to the vehicle-treated controls (* *p* < 0.05, two-tailed Student’s *t*-test). The mRNA expression of the 5-HT_2B_ and 5-HT_2C_ receptors in the agmatine-treated rats was not statistically different from that in the vehicle-treated controls (*p* > 0.05, two-tailed Student’s *t*-test).

## 3. Discussion

**Figure 6:**
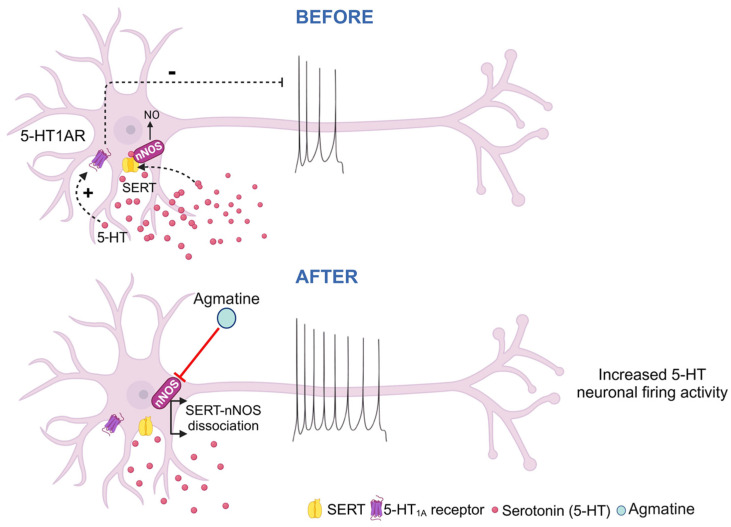
Effect of the acute agmatine administration on the excitability of 5-HT neurons; SERT, serotonin (5-HT) transporter; nNOS, neuronal nitric oxide synthase; 5-HT1AR, 5-HT_1A_ receptor.

We found that both the acute and chronic agmatine stimulated 5-HT neurons of the DRN. Chronic agmatine also enhanced the expression of 5-HT_1B_ and 5-HT_2A_ receptors in the DRN. The DRN expressions of the other subtypes of 5-HT receptors, belonging to the 5-HT_1_ and 5-HT_2_ subfamilies, as well as the expression of nNOS and SERT, were not affected by chronic agmatine treatment. The absence of significant changes in nNOS and SERT mRNA expression following chronic agmatine treatment, despite its robust effects on 5-HT neuronal firing, suggests that agmatine likely modulates serotonergic activity through functional interactions with the nNOS/SERT protein complex rather than through transcriptional regulation (Figure 6).

It was found that the acute administration of agmatine led to the activation of 5-HT neurons of the DRN (Figure 1). It is consistent with the inhibitory effect of the NO donor on 5-HT neurons, as reported in a previous study [15]. Since 5-HT neurotransmission is fundamental in antidepressant drug response [7], our findings may explain the antidepressant-like behavioral effect of acute agmatine, as reported in a previous study [13]. Since 5-HT stimulates BDNF expression [16], the excitation of 5-HT neurons by acute agmatine may explain its ability to enhance BDNF levels, reported in the same study. Our findings on agmatine’s bi-directional effects on 5-HT neuronal firing is consistent with the previous work showing that 5-HT_1B_ autoreceptor expression in the DRN can either decrease or increase anxiety-like behaviors depending on stress context, suggesting that the modulation of serotonergic signaling has complex state-dependent effects on emotional behavior [17].

The acute effect of agmatine on the excitability of the DRN 5-HT neurons was observed within seconds after the drug injection. It is therefore unlikely that any alteration in a protein and/or mRNA expression underlines the acute agmatine effect of the excitability of 5-HT neurons. It is however possible that the rapid dissociation of the nNOS/SERT dimer and subsequent 5-HT_1A_-autoreceptor-mediated disinhibition of 5-HT neurons is involved [9]. Further experiments should be performed to test this hypothesis. The relationship between agmatine’s inhibition of nNOS and enhanced 5-HT neuronal firing may involve complex NO-mediated pathways. Beyond its competitive inhibition of nNOS, agmatine also enhances NADPH oxidase activity of nNOS, leading to increased hydrogen peroxide production and subsequent oxidative inactivation of the enzyme through the alteration of its heme prosthetic group, which may contribute to agmatine’s overall effects on nitric oxide signaling [5]. Voltametric studies have revealed that NO plays a dual regulatory role in serotonergic function, with the microinjection of NOS inhibitors directly into the DRN specifically affecting certain aspects of serotonergic activity [18], while the extensive network of NO-synthesizing neurons projecting to the DRN from various brain regions suggests multiple pathways of modulation [19].

The mean spontaneous firing activity of 5-HT neurons in the agmatine-treated rats was significantly higher than in the vehicle-treated control (Figure 2). This finding was consistent with the previously observed antidepressant, anxiolytic, neuroprotective, and BDNF expression-enhancing effects of chronic agmatine [10,11,14]. It is also consistent with the previous finding that 5-HT neurons expressing nNOS have lower spontaneous firing activity than the non-nNOS expressing ones [15].

It is known that certain psychoactive drugs have distinct acute and chronic effects on the excitability of 5-HT neurons. The acute selective serotonin reuptake inhibitor (SSRI) escitalopram [20] or trace amine-associated receptor 1 (TAAR1) agonist RO5256390 [21] inhibits 5-HT neurons, while the chronic administration of these drugs has no effect on the firing rate of 5-HT neurons. It is also well established that adaptive changes in the expression and/or activity of the SERT and 5-HT_1A_ receptors are responsible for the different responses of 5-HT neurons to the acute and chronic administration of the same psychoactive drugs [7]. In the present study, the persistence of the ability of agmatine to stimulate 5-HT neurons after its chronic administration (Figure 2) was consistent with the lack of adaptive changes in the expression of the SERT and 5-HT_1A_ receptors after chronic treatment with this drug (Figure 3 and Figure 4).

It was found that chronic agmatine treatment led to an increased expression of the 5-HT_1B_ receptor-coding mRNA in the DRN (Figure 4), which is usually expressed within the cell bodies of 5-HT neurons. This mRNA was subsequently translated into the HT_1B_ receptor protein, which is expressed in the nerve terminals of 5-HT neurons in various brain areas, such as the hippocampus. These nerve terminal 5-HT_1B_ autoreceptors negatively regulate 5-HT neurotransmission [22]. It was, however, reported that the 5-HT_1B_ autoreceptor-mediated suppression of 5-HT release from the nerve terminal did not necessarily involve the suppression of the firing of 5-HT neurons. Furthermore, a 5-HT_1B_ agonist even increased the firing rate of 5-HT neurons, while a 5-HT_1B_ antagonist decreased the rate [23,24]. The increased expression of HT_1B_ autoreceptors, observed after chronic agmatine, does not therefore contradict the increase in the tonic firing activity of 5-HT neurons.

The observed upregulation of 5-HT_1B_ receptor expression following chronic agmatine treatment warrants careful interpretation. While this effect could be directly related to agmatine’s action, several alternative mechanisms should be considered. First, the chronic intravenous administration protocol itself may have introduced a stress component that could have influenced 5-HT_1B_ receptor expression, as previous studies have shown the stress-dependent modulation of serotonergic systems [17]. Additionally, the increased 5-HT_1B_ receptor expression may represent a compensatory homeostatic response to the enhanced serotonergic transmission induced by agmatine. Since 5-HT_1B_ receptor function as inhibitory autoreceptors on serotonergic terminals, their upregulation could serve as a negative feedback mechanism to maintain appropriate serotonin release. Furthermore, given that DRN serotonergic neurons receive substantial dopaminergic and glutamatergic inputs, agmatine’s effects on 5-HT_1B_ expression might be mediated indirectly through these neurotransmitter systems. The complex interplays between these various mechanisms deserve further investigation, particularly using selective antagonists and region-specific manipulations to dissect the relative contributions of direct and indirect pathways.

**Figure 7:**
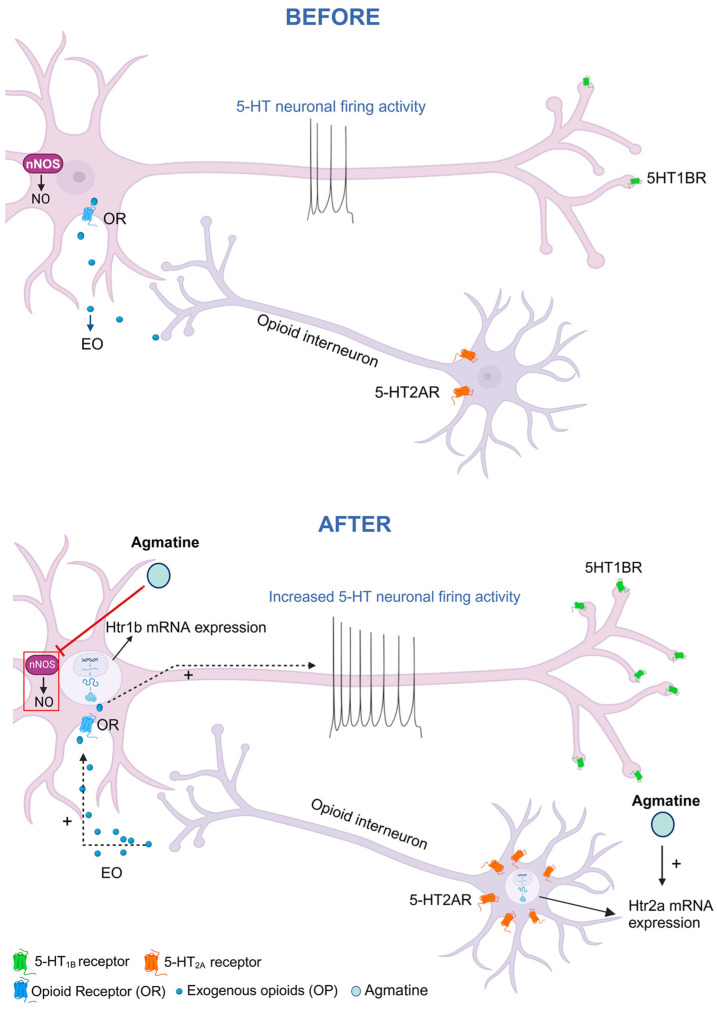
Effect of the chronic agmatine treatment on the excitability of 5-HT neurons; SERT, serotonin (5-HT) transporter; nNOS, neuronal nitric oxide synthase; 5-HT1BR, 5-HT_1B_ receptor; 5-HT2AR, 5-HT_2A_ receptor.

We found that sustained treatment with agmatine led to the increased expression of mRNA coding for 5-HT_2A_ receptors in the DRN (Figure 5). Since 5-HT_2A_ receptors are not known to act as autoreceptors, the 5-HT_2A_ receptor-coding mRNA detected in the DRN is likely to be expressed in the non-5-HT neurons, such as GABAergic and/or opioidergic interneurons [25,26]. It was reported that the local administration of 5-HT_2A/2C_ receptor agonist (+)-DOI hydrochloride (DOI) into the DRN activated local 5-HT neurons. Another study suggested that the 5-HT_2_ receptor-modulated stimulation of the DRN 5-HT neurons involved the excitation of opioidergic interneurons [25]. It is therefore possible that the chronic agmatine-induced stimulation of 5-HT neurons involves a 5-HT_2A_ receptor-based mechanism. The involvement of 5-HT_2A_ receptors in the beneficial CNS effect of agmatine has been previously reported as well. Santos and colleagues [27] found that the agmatine-induced antinociception in mice was significantly attenuated by 5-HT_2A_ receptor antagonist ketanserin. Freitas and co-authors [28] reported that ketanserin abolished the neuroprotective effect of agmatine in hippocampal neuronal cell culture. The ability of chronic agmatine to upregulate both 5-HT_1B_ and 5-HT_2A_ receptor expression while stimulating 5-HT neuronal firing aligns with the previous evidence that baseline serotonergic signaling may play a protective role against anxiety under normal conditions, though these relationships become more complex following stress exposure (Figure 7).

The primary limitation of this study is that the expression of 5-HT receptors was assessed in the DRN only. It is, however, known that the 5-HT_1A/1B_ receptors expressed in other brain areas, such as prefrontal cortex [29], are also involved in the regulation of excitability of the DRN 5-HT neurons. The effect of chronic agmatine on the nNOS, SERT, and 5-HT receptors in brain areas other than the DRN should be assessed in future studies.

## 4. Materials and Methods

### 4.1. Animals

Male Wistar rats (initial weight 250–350 g; 2–3 months old) were obtained from the Department of Toxicology and Laboratory Animals Breeding, Centre of Experimental Medicine of the Slovak Academy of Sciences, Dobra Voda, Slovak Republic. All the animals were housed (38 × 59 × 25 cm large cages) under standard laboratory conditions (temperature: 22 ± 2 °C, humidity: 55 ± 10%) with a 12 h light/12 h dark cycle (lights on at 7.00 a.m.). Pelleted food and tap water were available ad libitum. The State Veterinary and Food Administration of the Slovak Republic approved all the experimental procedures. The rats were handled according to the Directive 2010/63/EU of the European Parliament and of the Council of 22 September 2010 on the protection of animals used for scientific purposes.

### 4.2. Chemicals

Agmatine sulfate salt (A7127-5G, ≥97% purity) was purchased from Sigma-Aldrich (St. Louis, MO, USA). The DNA/RNA Shield (R1100-50, 50 mL) and Quick-RNA Microprep Kit (R1050) were obtained from Zymo Research (Irvine, CA, USA). The SOLIScript^®^ 1-step Multiplex Probe Kit was purchased from Solis Biodyne (Tartu, Estonia). Primers and probes were synthesized by MultiplexDX s.r.o. (Bratislava, Slovakia). All chemicals were of analytical grade and stored according to the manufacturer guidelines.

### 4.3. Electrophysiology

In vivo electrophysiological experiments were performed as previously described [21,30,31]. The rats were anesthetized with chloral hydrate (0.4 g/kg, i.p.) and mounted in the stereotaxic frame (David Kopf Instruments, Tujunga, CA, USA). Their scalp was opened, and a 3 mm hole was drilled in the skull for insertion of electrodes. Glass electrodes were pulled with a DMZ-Universal Puller (Zeitz-Instruments GmbH, Martinsried, Germany) to a fine tip of ~1 µM and filled with 2 M sodium chloride (NaCl). The impedance of the electrodes was 4–6 MΩ. The electrodes were lowered through the DRN using the hydraulic micro-positioner (David Kopf Instruments, Tujunga, CA). The action potentials generated by the neurons were recorded using the AD Instruments Extracellular Recording System (Dunedin, New Zealand). During the experiment, the rats’ body temperatures were maintained at 37 °C with a heating pad (Gaymor Instruments, Orchard Park, NY, USA). The 5-HT neurons of the DRN were identified according to the waveform of their action potential and the pattern of their generation, as explained in our previous works [21,30,31]. The neuronal activity was recorded and analyzed using the LabChart software, version 7 (AD Instruments, Dunedin, New Zealand). The burst activity characteristics were calculated using the burstiDAtor software (Nikolaas N. Oosterhof, Trento University, Trento, Italy; URL www.github.com/nno/burstidator; accessed on 23 March 2025). The onset of a burst was signified by the occurrence of two spikes with an interspike interval (ISI) < 0.01 s. The termination of a burst was defined as an ISI > 0.010 s for 5-HT neurons [32].

### 4.4. Assessment of the Acute Agmatine Effect on 5-HT Neuronal Firing Activity

In the experiments aiming to assess the effect of acute agmatine on the firing activity of 5-HT neurons, after a first DRN 5-HT neuron with a stable firing activity was found, its basal firing was recorded for two minutes. Thereafter, 1 mg/kg of agmatine was administered via a catheter placed in the femoral vein. The neuronal firing activity was recorded for another two minutes. Subsequently, an additional dose of 1 mg/kg of agmatine was administered (the cumulative dose 2 mg/kg), and the neuronal firing activity was recorded for another two minutes. Finally, the last administration of 1 mg/kg of agmatine was performed (the cumulative dose 3 mg/kg), and the neuron was recorded for the last two minutes. Thereafter, the animal was euthanized by an overdose of chloralhydrate.

### 4.5. Assessment of the Chronic Agmatine Effect on 5-HT Neuronal Firing Activity

In experiments aiming to assess the effect of chronic agmatine on the firing activity of 5-HT neurons, the rats were randomly divided into two groups. One group received daily injections of agmatine (40 mg/kg/day, i.p.). The control animals received daily i.p. injections of the vehicle (saline: 0.9% sodium chloride: NaCl in water). On the 15th day, electrophysiological experiments were performed. The electrode was lowered through the DRN 3–4 times and the spontaneously active 5-HT neurons detected during each electrode descent were recorded. After the completion of the electrophysiological recording, the rats were euthanized by an overdose of chloralhydrate.

### 4.6. Assessment of Target Gene Expression in DRN Using Multiplex RT-qPCR

Following the electrophysiological recordings, the rats were euthanized by decapitation, and their brains were rapidly removed. The fresh brains were placed on a cold plate and sectioned coronally with a razor blade. The DRN region was precisely isolated from these coronal sections using a custom-made punch tool (1.5 mm diameter) and transferred to 1.5 mL tubes. The samples were immediately flash-frozen in liquid nitrogen and stored at −80 °C until further analysis. On the day of the PCR experiments, DNA/RNA Shield solution (300 µL; Zymo Research) was added to the samples immediately upon removal from −80 °C storage to prevent RNA degradation. Total RNA was extracted using the Quick-RNA Miniprep Kit with DNase I treatment (Zymo Research) according to the manufacturer’s protocol.

The RT-qPCR primer and probe sequences were designed for multiplex analysis. The primers and dual-labeled hydrolysis probes were obtained from MultiplexDX s.r.o. (Bratislava, Slovakia). The sequences are detailed in Table 1. Each probe was labeled at the 5′ end with a fluorescent reporter dye (FAM, HEX, ROX and Cy5) and at the 3′ end with a quencher dye (BHQ1, BHQ2). BLAST analysis (https://blast.ncbi.nlm.nih.gov/Blast.cgi; accessed on 23 March 2025) confirmed the specificity of the primer/probe sets to their target sequences.

The first multiplex reaction contained 5-HT_1A_, 5-HT_2A_, nNOS, and GAPDH. The second multiplex reaction contained 5-HT_1B_ and GAPDH. The third multiplex reaction contained 5-HT_2B_, 5-HT_2C_, SERT, and GAPDH. For the thermal cycle reactions, a CFX96 Real-Time System (Bio-Rad Laboratories, Hercules, CA, USA) was used. For the first and third multiplex reactions, the thermal cycling conditions were as follows: cDNA synthesis at 50 °C for 30 min, then initial denaturation at 95 °C for 10 min, followed by 40 cycles of denaturation at 95 °C for 15 s, and combined annealing/extension at 60 °C for 1 min with a plate read. For the second multiplex reaction, the protocol was modified with an annealing temperature of 56 °C for 1 min during the cycling steps, while maintaining the same initial cDNA synthesis and denaturation conditions. Gene expression was analyzed using CFX Maestro Software version 1.1 (Bio-Rad Laboratories, CA, USA) using the 2^−ΔΔCt^ method, with GAPDH serving as the reference gene. Relative expression values were calculated and normalized to the control samples.

### 4.7. Statistical Analysis

To analyze the effect of different doses of acute agmatine on 5-HT neuronal firing activity, a one-way repeated-measures ANOVA (RM ANOVA) was performed with time as a factor of comparison (baseline and after administration of 1, 2, and 3 mg/kg of agmatine), followed by the Bonferroni post hoc test. To assess the effect of chronic agmatine on 5-HT neuronal firing rate and the expression of NOS, SERT, 5-HT_1A/1B_, and 5-HT_2A/2B/2C_ mRNA in the dorsal raphe nucleus (DRN), a two-tailed Student’s *t*-test was used to compare agmatine-treated and vehicle-treated groups. The PCR data were analyzed using GraphPad Prism 10, and the statistical significance was determined using a two-tailed Student’s *t*-test. Results are expressed as mean ± standard error of the mean (SEM), and statistical significance was set at *p* ≤ 0.05.

## 5. Conclusions

We found that acute and chronic treatment with agmatine led to the stimulation of 5-HT neurons of the DRN. The ability to stimulate central 5-HT neurons might explain the anxiolytic and antidepressant-like effects of agmatine observed in the previous studies. While the acute effect of agmatine is likely to be based on its direct effect on the nNOS-SERT complex, the chronic effect of this drug putatively involves the upregulation of the 5-HT_2A_ receptor. Since the lack of a timely and adequate response to antidepressant drugs frequently results from the auto-inhibition of 5-HT neurotransmission, the ability of the nNOS inhibitors to stimulate 5-HT neurotransmission may make them potential antidepressants on their own and/or as adjuncts to other antidepressants, such as SSRIs and/or TAAR1 agonists. On the other hand, a chronic agmatine-induced increase in the expression of 5-HT_1B_ autoreceptors might have a diminishing effect on the net 5-HT transmission. The exact effect of nNOS inhibition on the nerve terminal 5-HT release should be examined in future studies.

## Figures and Tables

**Table 1:** Forward and reverse primers and probe sequences used for RT-qPCR analysis of target genes.

Target Gene	Forward	Reverse	Probe
5-HT_1A_	5′-TGG GTA CTC TCA TTT TCT G-3′	5′-CAG CAC TGA TAC CAT GAG-3′	FAM-TCA GTA ACC GCC AAG GAG CC-BHQ_1
5-HT_2A_	5′-CAG AGT TCT CTG TCA TCA-3′	5′-GCA CCA CAT TAC AAC AAA-3′	HEX-TCC AAC GGT CCA TCC ACA GAG-BHQ_1
nNOS	5′-GGC GAA CAA CTC CCT CAT TA-3′	5′-TTG GAA AGA CCT TGG GTC AG-3′	ROX-TCC TCT TCC AGC TTC GGT CAT TGC-BHQ_2
5-HT_1B_	5′-GGT GGA CTA TTC TGC TAA A-3′	5′-GGA GTA GAC CGT GTA GAG-3′	FAM-AAG CAG TCC AGC ACC TCC TC-BHQ_1
5-HT_2B_	5′-CTG TGT CCT GCC TGG TTA TT-3′	5′-GCA CTG ATT GGC CTG AAT TG-3′	FAM-TGT GCC ATT TCC CTG GAT CGC TAT-BHQ_1
5-HT_2C_	5′-GCA CGG GAC TGT GAT GTT AT-3′	5′-GTA GAT GAG CAG AGC GAG AAT C-3′	HEX-ACA CCT AAA TGG ACA GAT TCA GTG GCA-BHQ_1
SERT	5′-CAC CGT AAT CTA CTT TAG C-3′	5′-GAC AGA GAG GAC AAT GTA-3′	ROX-CCA CAC CAC CTT GCC AGA TG-BHQ_2
GAPDH	5′-CTC CCT GTT CTA GAG ACA-3′	5′-CCA TGT AGT TGA GGT CAA-3′	Cy5-CAG TGC CAG CCT CGT CTC ATA-BHQ_2

## Data Availability

The data that support the findings of this study are available from the corresponding authors upon reasonable request.

## Supplementary Materials

The following supporting information can be downloaded at: https://www.mdpi.com/article/10.3390/ijms26073087/s1.

## Abbreviations

The following abbreviations are used in this manuscript:

5-HT	Serotonin
ANOVA	Analysis of variance
NO	Nitric oxide
NOS	Nitric oxide synthase
nNOS	Neuronal nitric oxide synthase
MDPI	Multidisciplinary Digital Publishing Institute
SERT	5-HT transporter
SSRIs	Selective serotonin reuptake inhibitors
TAAR1	Trace amine associated receptor 1
