# Supplementary figures and images for: Agmatine Enhances Dorsal Raphe Serotonergic Neuronal Activity via Dual Regulation of 5-HT1B and 5-HT2A Receptors
Source: Int J Mol Sci. 2025 Mar 27;26(7):3087. doi: 10.3390/ijms26073087 (PMC11988524; doi:10.3390/ijms26073087)

5HT1B

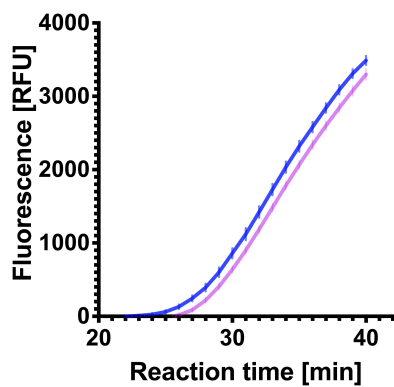

5HT2A

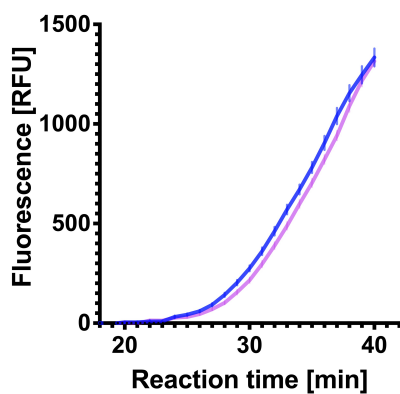

5HT1A

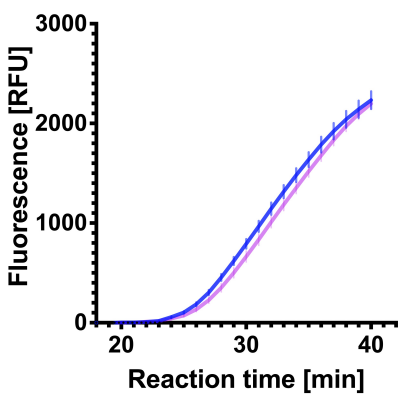

5HT2B

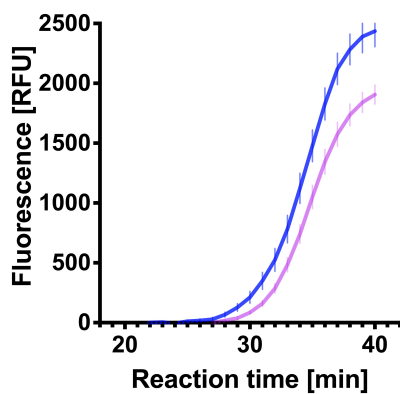

nNOS

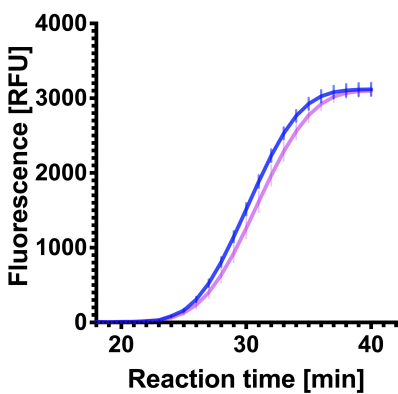

5HT2C

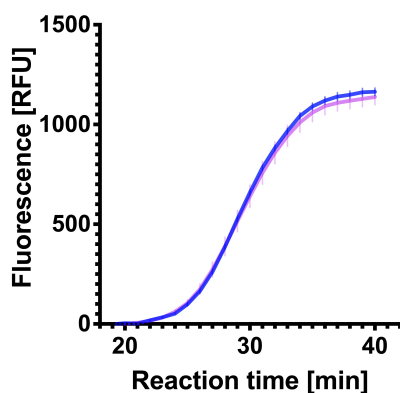

SERT

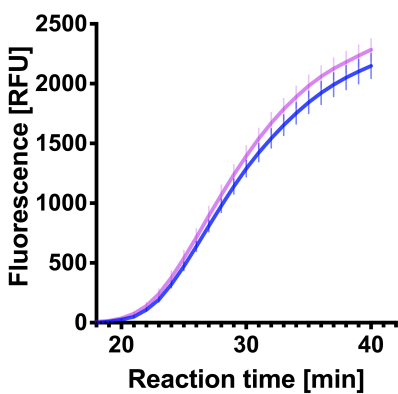

— Agmatine  
— Control

Supplement: Supplementary file 1 [file ijms-26-03087-s001.zip › ijms-3515675-supplementary.pdf]
